# P02.133. Randomized double blinded clinical trial of Ojeoksan products extracted through different methods for low back pain

**DOI:** 10.1186/1472-6882-12-S1-P189

**Published:** 2012-06-12

**Authors:** S Lee, J Lee

**Affiliations:** 1 Kyunghee University, Daejeon, Republic of Korea

## Purpose

The aim of the present study is to observe the efficacy of Ojeoksan and the difference in the results induced by extracting methods among mixtures of independently extracted herbs (MIE group), extract from combined decoction (ECD group) and placebo (Placebo group).

## Methods

The study was approved by the Ethics Committee of Kyunghee Oriental Medical Center in Seoul, Korea. Sixty subjects (M:F=26:34) with low back pain were recruited in the study and randomly allocated to MIE group, ECD group and Placebo group. During 4 weeks, the MIE group took the Ojeoksan Mixture of Independently Extracted Herbs, the ECD group took the Ojeoksan Extract from combined Decoction, and the Placebo group took a placebo. The effect of Ojeoksan on pain was measured with VAS (Visual Analogue Scale) and SF-36 Bodily Pain scale. Disability was measured with RMDQ (Roland-Morris Disability Questionnaire). General health was measured with the SF-36 (36-Item Short-Form Quality of Life Questionnaire) and range of motion was measured with MMST (Modified-Modified Schober Test) at baseline, 2 weeks and 4 weeks. Efficacy was evaluated with the SPSS 12.0 paired t-test for intragroup testing and ANCOVA (Analysis of covariance) for intergroup testing.

## Results

After 4 weeks, the MIE group showed significant improvement on VAS, RMDQ and MMST; the ECD group showed significant improvement on VAS, SF-36 Bodiliy Pain scale, RMDQ, SF-36 and MMST; the Placebo group showed significant improvement on VAS and MMST by paired t-test. In RMDQ, each group showed a significant difference, but other scales showed no difference analysed by ANCOVA.

## Conclusion

According to the results, Ojeoksan is more efficacious on pain, disability and general health for low back pain patients than placebo. Ojeoksan extract from combined decoction is more efficacious than Ojeoksan mixture of independently extracted herbs and placebo on disability caused by lumbar back pain.

